# Evaluation of the usefulness of saliva for mosaic loss of chromosome Y analysis

**DOI:** 10.1038/s41598-021-83308-8

**Published:** 2021-02-12

**Authors:** Tsuyoshi Hachiya, Takuro Kobayashi, Wataru Tsutae, Pamela Hui Peng Gan, Iri Sato Baran, Shigeo Horie

**Affiliations:** 1 Department of Advanced Informatics for Genetic Disease, Graduate School of Medicine, Juntendo University, Tokyo, Japan; 2 Department of Urology, Graduate School of Medicine, Juntendo University, 3-1-3 bunkyo-ku, Hongo, Tokyo, 113-8421 Japan; 3 Genesis Healthcare Co, Tokyo, Japan

**Keywords:** Genetic association study, Biomarkers

## Abstract

Mosaic loss of chromosome Y (mLOY) in leukocytes has attracted much attention as an emerging biomarker of aging and aging-related diseases. We evaluated the usefulness of saliva for mLOY analysis and showed that saliva-derived mLOY is significantly associated with aging and increased physical activity, but not with smoking. While these data support the robust association between saliva-derived mLOY and aging, caution is required when comparing data from saliva-derived and blood-derived mLOY.

## Introduction

The human genome changes over a person’s lifetime, including the attrition of telomere length and the accumulation of somatic mutations, even in non-cancerous cells^1^. As a consequence of somatic mutations occurring in stem cells, a detectable clonal population of cells harbor a postzygotic mutation that is distinct from inherited germline variation^2,3^. Among such somatic mutations, a commonly detected structural event in aging males is mosaic loss of the Y chromosome (mLOY)^3^; this refers to the loss of the entire Y chromosome in a subset of cells, while the remainder of cells retain a normal Y chromosome^4,5^.

Accumulating evidence suggests that presence of mLOY in leukocytes is associated with aging-related diseases^6^. The prevalence of detectable mLOY in leucocytes increases as a function of age^4^ and is associated with all-cause mortality^7,8^. Cigarette smoking is associated with an elevated prevalence of mLOY^4,9^. Findings from epidemiological studies show associations of mLOY in leukocytes and a risk of non-communicable diseases, including cancer^3,6,7^, diabetes mellitus (DM)^8^, and Alzheimer’s disease^10^. Genetic investigations have elucidated that mis-segregation during mitosis, cell-cycle dysregulation, and enhanced genomic instability may be involved in the aetiology of mLOY^11–13^. It is noteworthy that levels of evidence for these previously-reported associations were varied. Associations of mLOY with chronological age and cigarette smoking were robustly observed in multiple studies even after careful adjustments for potential confounding factors^4,8,9,11^. Association between mLOY and cancer risk was found and replicated in large-scale studies^3,7,11^, but the association was moderate after an adjustment for smoking^3^. In another study, a null association was reported between smoking and mLOY^4^. Other associations were reported in only one or a few studies^8,10^.

Recent studies have highlighted discrepancies in findings from mLOY derived from blood compared to other tissues^14,15^. In elderly men (over 90 years old), mLOY was observed less frequently in buccal cells than in leukocytes^14^. In another study, the association of mLOY with age and smoking was observed in both buccal and blood samples; however, the strength of this association was significantly weaker in buccal compared to blood samples^15^.

The aim of this study was to evaluate the usefulness of saliva for mLOY analysis. Collecting saliva samples is less invasive than obtaining blood samples^16^. Saliva samples include buccal epithelial cells and leukocytes, with composition varying among subjects^16^. Saliva yields DNA of sufficient quantity and quality to compare favorably with blood as a source of DNA for genetic research^17^. Whether saliva-derived genetic data can be used for mLOY analysis is a key issue for mLOY studies performed in cohorts from large populations.

## Results and discussion

We analysed saliva-derived genetic data from 25,121 men recruited in Japan, and investigated the association of saliva-derived mLOY with age, smoking, alcohol drinking, body mass index (BMI), and physical activity. This sample size provided adequate power to detect statistical significance (Supplementary Table 1). mLOY was detected from fluorescence intensity data of > 2400 microarray probes on the male-specific region of chromosome Y. To enable comparisons with existing evidence of mLOY in blood, we defined detectable mLOY as a previously described threshold value of -0.15 for the median of log R ratios for probes in the male-specific region of chromosome Y (mLRR-Y)^8^.

**Figure 1:**
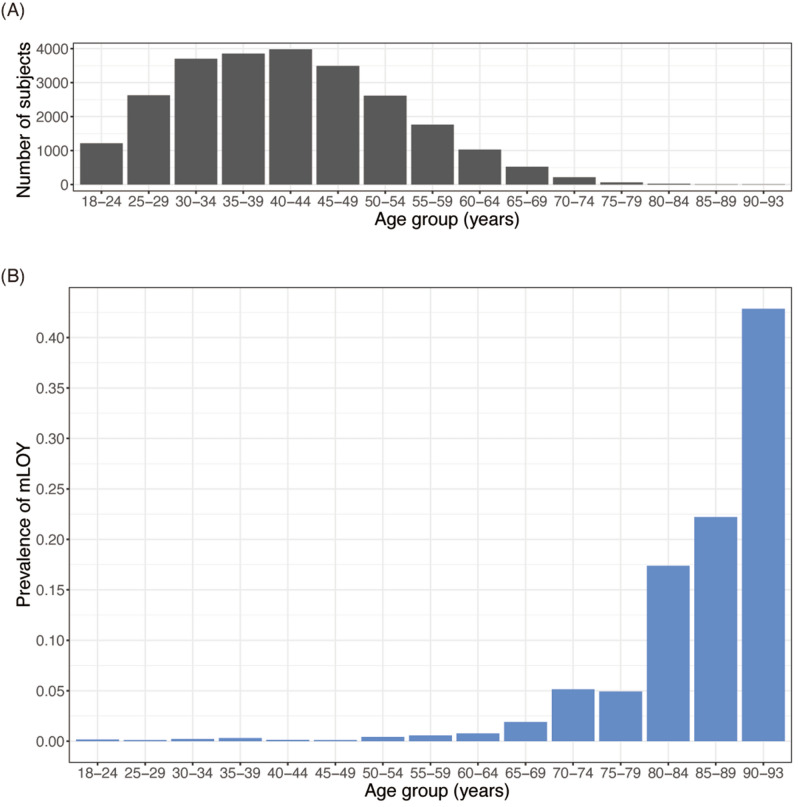
Number of study participants and prevalence of mLOY according to age group. The *x*-axis indicates age group (years). (**A**) The *y*-axis shows the number of study participants. (**B**) The *y*-axis shows the prevalence of mLOY. mLOY indicates mosaic loss of the Y chromosome.

The mean (± standard deviation) age of men at saliva collection was 41.7 ± 11.6 years (range 18–93 years; Fig. 1A). mLOY was detected in 96 subjects. The prevalence of mLOY in saliva increased steeply with age for subjects ≥ 65 years (Fig. 1B). The prevalence of mLOY was 5.14% (age group: 70–74 years), 17.4% (age group: 80–84 years), and 42.9% (age group: 90–93 years; Supplementary Table 2). The association between mLOY in saliva and age group was highly significant (*P* for trend = 2.8 × 10^–29^). The prevalence of mLOY in saliva according to age group was similar to data previously reported for blood^4,8,14^.

**Figure 2:**
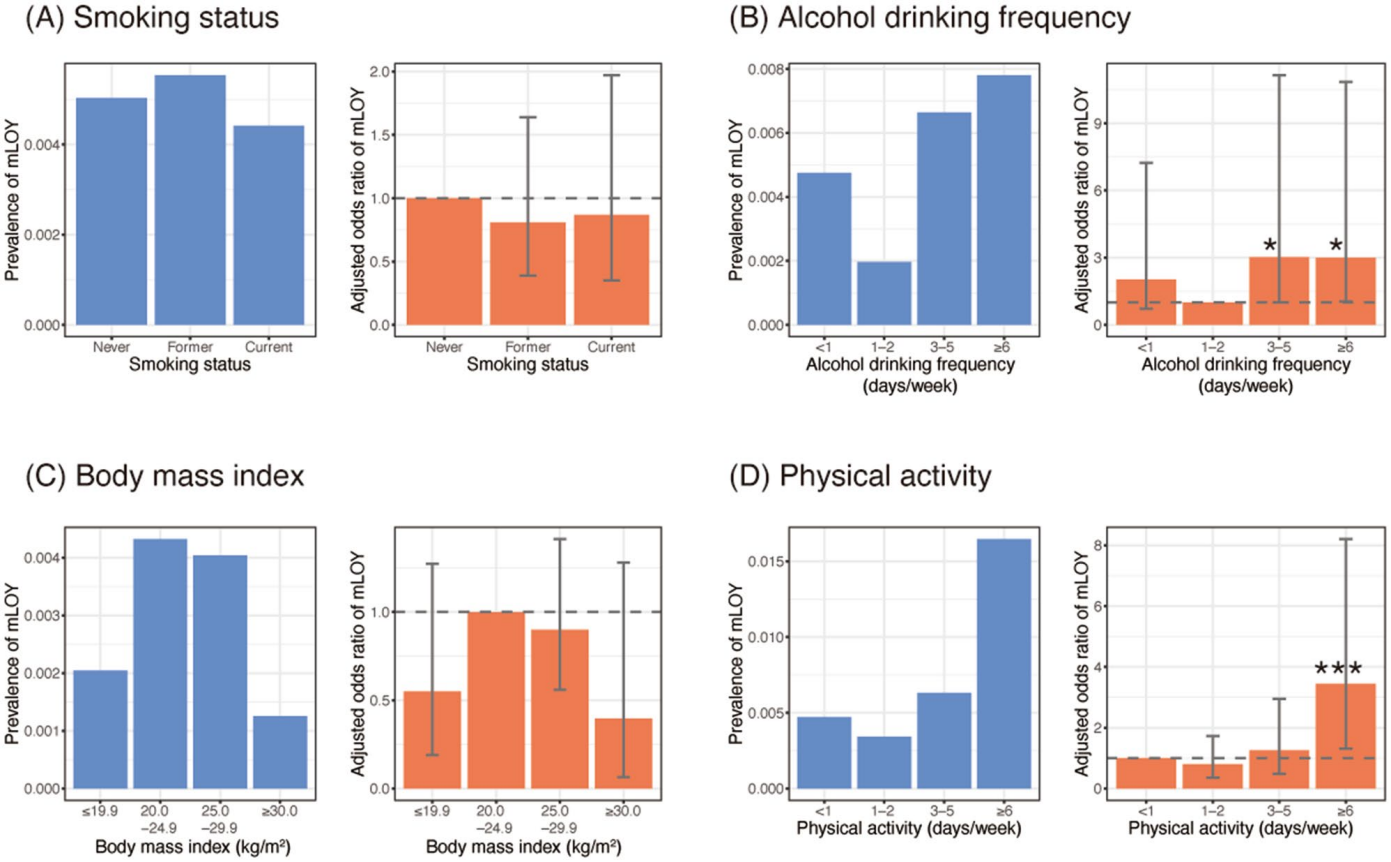
Association of mLOY with lifestyle factors. The left panel shows the prevalence of mLOY, and the right panel displays the odds ratio of mLOY adjusted for age group. Error bars in the right panel indicate 95% confidence interval of odds ratio: (**A**) smoking status, (**B**) alcohol drinking frequency, (**C**) body mass index, and (**D**) physical activity. mLOY indicates mosaic loss of the Y chromosome. *P < 0.1, ***P < 0.01.

The prevalence of mLOY in saliva in never-smokers was 0.50%, which was not significantly different to current smokers (0.44%; *P* = 0.75) and former smokers (0.55%; *P* = 0.55) after adjustment for age (Fig. 2A). This finding was not consistent with previous studies for mLOY in blood; the risk of mLOY in blood was ~ 3-hold higher in current smokers than in never-smokers^4,8,9,11^.

The prevalence of mLOY in frequent alcohol drinkers (3–5, and ≥ 6 days per week) was > threefold higher than in occasional alcohol drinkers (1–2 days per week), and differences were marginally significant after adjustment for age (*P* = 0.06 for both 3–5 days/week and ≥ 6 days/week; Fig. 2B). In a previous study, the prevalence of mLOY in blood was modestly higher in frequent drinkers (> 3 days per week) than in occasional drinkers (1–3 days per week) (*P* = 0.0005)^8^. The relationship between mLOY and alcohol drinking appears to be similar for saliva and blood samples.

The prevalence of mLOY in saliva was slightly lower in lean (BMI < 20 kg/m^2^) and obese (BMI ≥ 30 kg/m^2^) subjects, compared to subjects who had a standard BMI (20.0–24.9 kg/m^2^), although these differences were not significant after adjustment for age (Fig. 2C). A previous study showed that obesity was associated with a decreased prevalence of mLOY in blood (*P* = 0.0001)^8^.

The prevalence of mLOY increased with the frequency of physical activity (*P* for trend = 0.04). Compared to sedentary subjects (physical activity < 1 day per week), subjects with frequent physical activity (≥ 6 days per week) had a 3.45-fold higher chance of detectable mLOY (95% confidence interval, 1.30–8.21; *P* = 0.007 after adjustment for age; Fig. 2D). Interestingly, frequent physical activity was significantly associated with mLOY in men ≥ 60 years old (*P* = 0.02), but not in men < 40, 40–49, or 50–59 years old (Supplementary Table 3). Regular physical activity is associated with a lower risk of mortality^18^; conversely, excessive exercise can have an adverse effect on the immune and endocrine systems^19,20^. Detectable mLOY in elderly men who undertake frequent physical activity may, therefore, be detrimental; however, this hypothesis needs to be further explored. The association between physical activity and blood-derived mLOY has not been shown to be significant^8^. Further studies are warranted to determine if this discrepancy is due to differences in blood- and saliva-derived mLOY, or other reasons, such as different ethnicities of study populations^8,11^.

Due to relatively small number of patients and insufficient statistical power, we were unable to examine the association of mLOY in saliva with risk of diseases, such as cancer and DM (Supplementary Table 4).

Both buccal epithelial cells and leukocytes are included in saliva. Associations of mLOY with chronological age and smoking have been robustly observed in both buccal and blood tissues^4,9^, although the associations were stronger in blood than in buccal samples^14,15^. With a simplistic point of view, the association between saliva-derived mLOY and age or smoking would be similar to those observed in buccal and blood. As expected, mLOY in saliva was robustly associated with chronological age, and the prevalence of mLOY according to age groups seemed to be similar to that reported in blood-based previous studies^4,8,14^. Contrary to expectations, saliva-derived mLOY was not significantly associated with smoking in the present study. Cigarette smoking affects leukocyte subpopulations in peripheral blood; for example, current smokers had a higher proportion of CD4^+^ lymphocytes and neutrophils than never smokers^21^. Although little is known about the effect of cigarette smoking on cell composition in saliva, long-term smoking was associated with decreased secretion of saliva^22^. Thus, smoking could influence salivary cell composition and fluctuations in cell composition might weaken the association between mLOY and smoking. The sample size of the present study had an adequate power (95.2%) for the comparison between current and never smokers when assumed an odds ratio of 3.1, which is a reported odds ratio of the association between blood-derived mLOY and smoking^15^. However, the statistical power was modest (42.0%) when assumed an odds ratio of 1.8, which is a reported odds ratio of the association between buccal-derived mLOY and smoking^15^. Accordingly, our data indicated that the association between mLOY and smoking is considerably weaker in saliva than in blood, or that saliva-derived mLOY is not at all associated with smoking. Further studies incorporating a larger number of elderly subjects are needed to distinguish the two possibilities.

There are several limitations in the present study. Most of the study subjects were younger than age ranges where mLOY is frequently observed, and therefore, mLOY was detected in a limited number of subjects (*n* = 96). This might raise concern about statistical power to robustly detect significant difference between mLOY events and lifestyle factors. Although our power calculation showed that the present study had an adequate power, incorporation of more elderly subjects in further studies would be important to confirm our findings. In addition, we did not directly compare saliva- and blood-derived mLOY signals from the same individuals. Instead, we investigated the association between saliva-derived mLOY and lifestyle factors, and compared the saliva-derived association results with previously-reported blood-derived association results. Thus, we should consider several possibilities when the association results were inconsistent between saliva and blood. First, the inconsistency may be attributable to *bona fide* difference between saliva- and blood-derived mLOY signals. Second, insufficient statistical power may lead to the inconsistency. Third, differences in ethnical, demographic, sociological, or other background factors may contribute to the inconsistency. Accordingly, we carefully interpreted the inconsistency between saliva- and blood-derived association results in the present study.

In summary, this study showed that the prevalence of mLOY in saliva-derived DNA increased with age. The associations of mLOY with alcohol drinking, and BMI were similar for saliva and blood, while the association of mLOY with smoking differed considerably between saliva- and blood-derived DNA. A novel association was also found for mLOY and frequent physical activity in men ≥ 60 years old. In conclusion, saliva-derived mLOY is associated with age, but caution is required when comparing and interpreting data from saliva-derived mLOY and blood-derived mLOY.

## Methods

### Study subjects and genotyping

We used data collected from a direct-to-customer genetic testing service, GeneLife (Genesis Healthcare Co., Tokyo, Japan). Saliva samples were collected using Oragene DNA Collection Kit (DNA Genotek Inc., Ottawa, Ontario, Canada) or Zeesan Saliva DNA Sample Collection Kit (Zeesan Biotech Co., Ltd., Xiamen, China), and DNA was extracted using Agencourt DNAdvance (Beckman Coulter, Inc. CA, USA). Genotyping was performed using Infinium CoreExome-24 + kit (Illumina Inc., San Diego, CA, USA) in three separate batches due to minor modifications in custom markers (referred to as Genesis Healthcare customized chip version 4.0, 4.1 or 4.2). The participants were asked to complete a self-administered, internet-based questionnaire covering tobacco smoking, alcohol drinking, physical activity, body height and weight, and medical history.

This study was conducted with the approval of the Institutional Review Board at Juntendo University and Institutional Review Board at Genesis Healthcare Co. All participants provided written informed consent. This study was conducted according to the principles expressed in the Declaration of Helsinki 2013.

### Definition of mLOY

Fluorescence intensity data of microarray probes mapped on the male-specific region of chromosome Y (56-Mb region between pseudoautosomal regions 1 and 2) were used to detect mLOY as previously described^4,7^. Briefly, fluorescence intensity data was normalized and log-transformed using Illumina GenomeStudio software. The normalized and log-transformed intensity data was referred to as “log R ratio”, as in the literature^7^. Missing data entry of the log R ratio indicated low measurement quality, possibly due to low input DNA amount or female-derived DNA. We excluded subjects who had missing data entries in > 1% of the probes for the male-specific region in order to filter out low quality intensity data as well as to ensure that all samples included in our analyses were derived from males. The median of log R ratios for probes in the male-specific region of chromosome Y (mLRR-Y) was calculated for each subject, and we defined mLRR-Y ≤ −0.15 as detectable mLOY. The same threshold was used in previous studies^4,8,15^, and we applied it for comparison to mLOY in blood.

The number of markers in the male-specific region of chromosome Y was 2464, 2601, and 2623 in the chip version 4.0, 4.1, and 4.2, respectively. Ranges of experimental noise in mLRR-Y were estimated for each batch, and we confirm that the above-mentioned threshold (mLRR-Y ≤ −0.15) was not included in the range of experimental noise for all three batches (Supplementary Figs. 1, 2, 3), indicating that the threshold applied in this study was appropriate from an experimental noise perspective. We showed an exemplified plot of the probe location on the chromosome Y and log R ratio values for a subject genotyped by the chip version 4.2 (Supplementary Fig. 4), indicating that almost probes on the chromosome Y were located within the male-specific region (2,623 probes were located within the male-specific region, whereas 181 probes were located outside the male-specific region).

### Statistical analysis

The association of mLOY with age group was assessed using logistic regression analysis with no adjustment for variables. The associations of mLOY with smoking, alcohol drinking, BMI, and physical activity were evaluated using logistic regression analysis with adjustment for fifteen age group categories (18–24, 25–29, 30–34, 35–39, 40–44, 45–49, 50–54, 55–59, 60–64, 65–69, 70–74, 75–79, 80–84, 85–89, or 90–93 years). Two-sided *P* < 0.05 was considered statistically significant, and two-sided *P* < 0.1 was considered marginally significant in all analyses. Power was estimated based on an equation proposed by Hsieh et al.^23^, which is implemented in the ‘powerLogisticBin’ function of the ‘powerMediation’ package in R. All statistical analyses were performed using R software, version 3.6.1 (R Foundation for Statistical Computing, Vienna, Austria).

## Supplementary Information

Supplementary Information.

## Data Availability

Reasonable requests for data supporting the findings from this study are available from the corresponding author.
