# Supplementary material for: Evaluation of the usefulness of saliva for mosaic loss of chromosome Y analysis
Source: Sci Rep. 2021 Feb 12;11:3769. doi: 10.1038/s41598-021-83308-8 (PMC7881200; doi:10.1038/s41598-021-83308-8)
Supplement: Supplementary file 1 — Supplementary Information. [file 41598_2021_83308_MOESM1_ESM.docx]

**Supplementary information for ‘Evaluation of the usefulness of saliva for mosaic loss of chromosome Y analysis’**

Supplementary Figures S1 to S4

Supplementary Tables S1 to S4

**
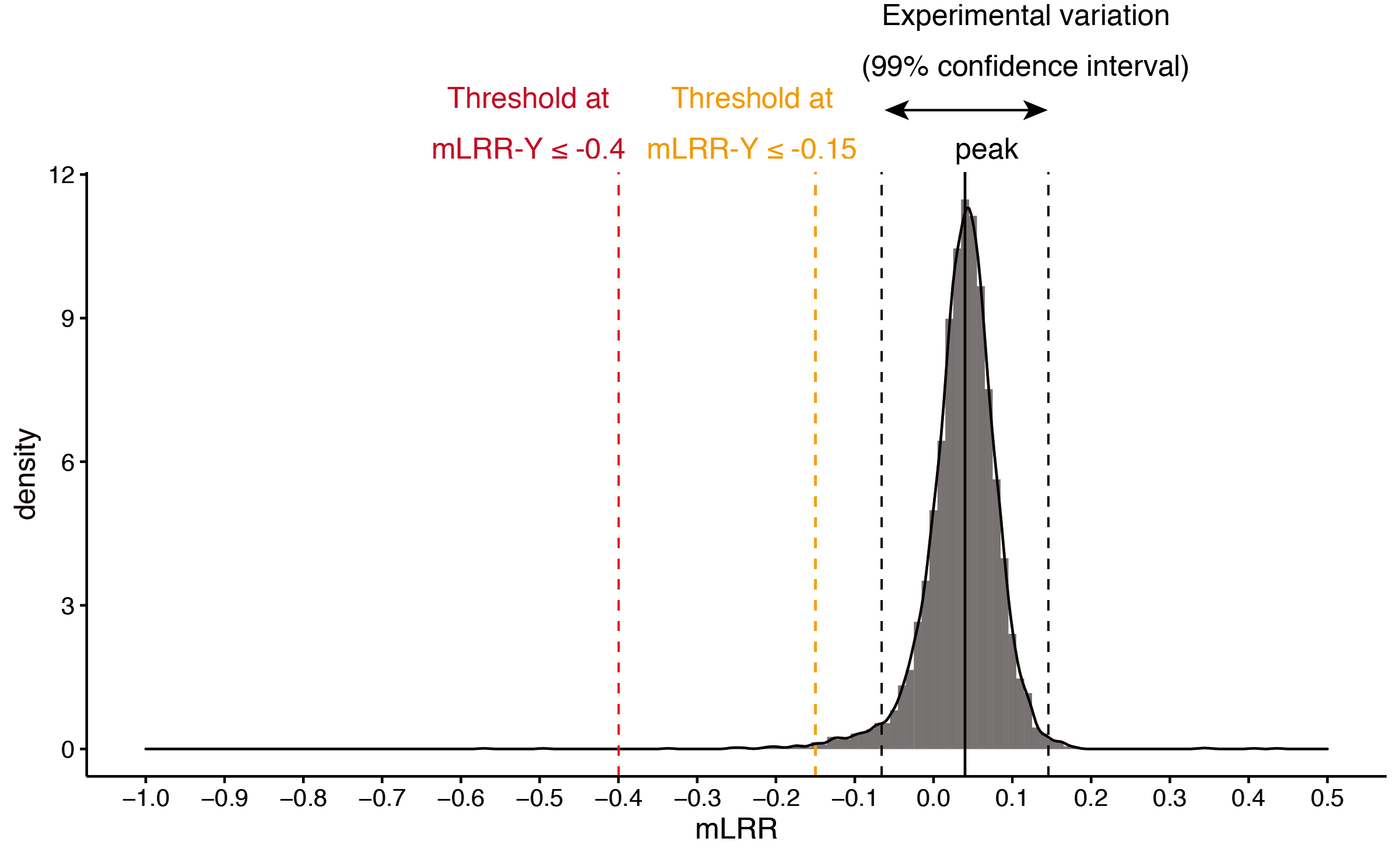
**

**Supplementary Figure 1. Distribution of mLRR for the chip version 4.0**

The *x*-axis indicates the median of log R ratios for probes in the male-specific region of chromosome Y (mLRR-Y). The *y*-axis shows the density of subjects genotyped by the Genesis Healthcare customized chip version 4.0 (*n* = 5,576). The number of markers in the male-specific region of chromosome Y was 2,464. The peak of the distribution is shown as a black vertical line, and ranges of experimental noise (99% confidence interval) are indicated by dashed black vertical lines. Thresholds at mLRR-Y ≤ -0.15 and ≤ -0.4 are shown as orange and red dashed lines, respectively. The threshold of mLRR-Y ≤ -0.15 was applied for all analyses in this study.

**
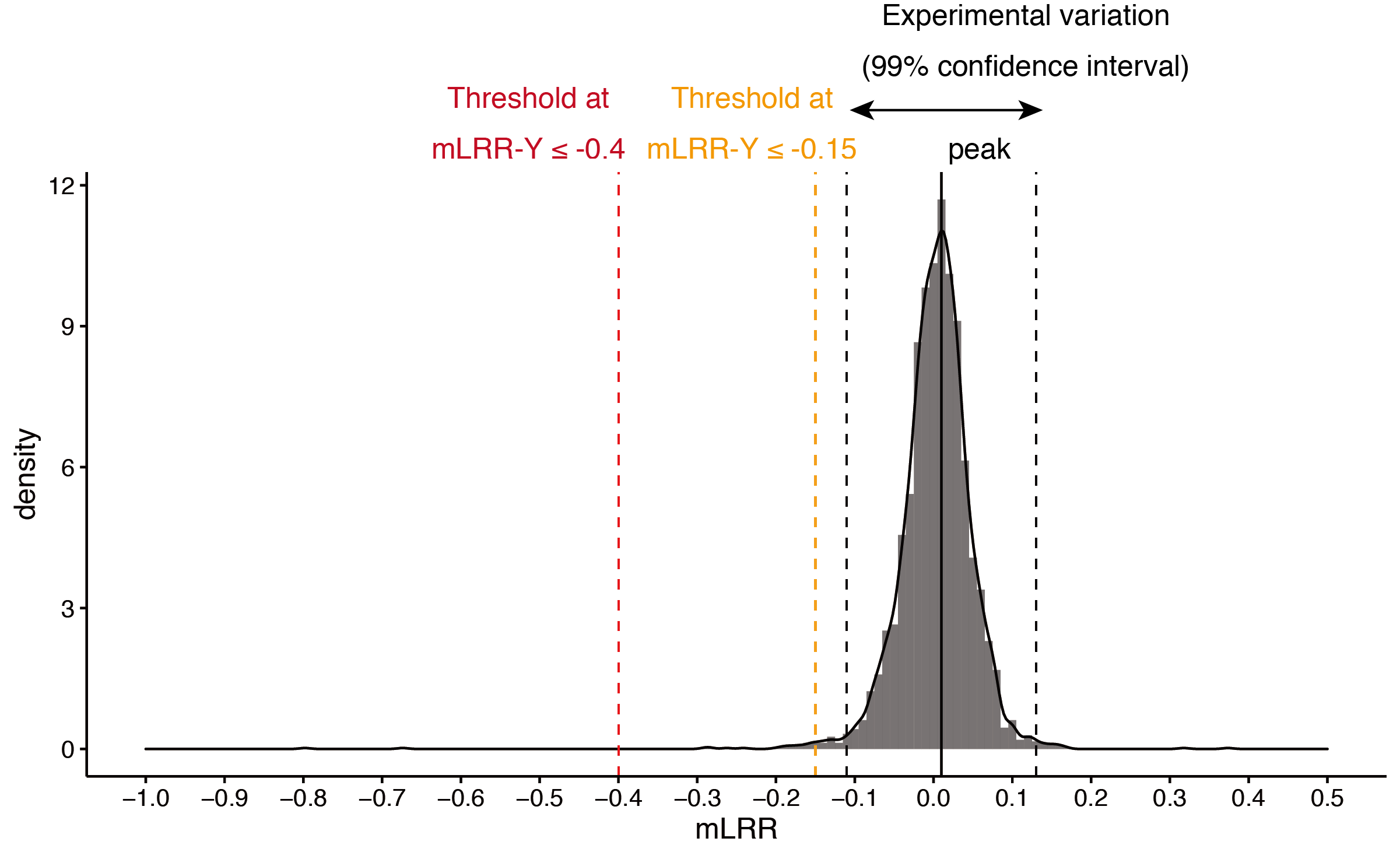
**

**Supplementary Figure 2. Distribution of mLRR for the chip version 4.1**

The *x*-axis indicates the median of log R ratios for probes in the male-specific region of chromosome Y (mLRR-Y). The *y*-axis shows the density of subjects genotyped by the Genesis Healthcare customized chip version 4.1 (*n* = 3,095). The number of markers in the male-specific region of chromosome Y was 2,601. The peak of the distribution is shown as a black vertical line, and ranges of experimental noise (99% confidence interval) are indicated by dashed black vertical lines. Thresholds at mLRR-Y ≤ -0.15 and ≤ -0.4 are shown as orange and red dashed lines, respectively. The threshold of mLRR-Y ≤ -0.15 was applied for all analyses in this study.

**
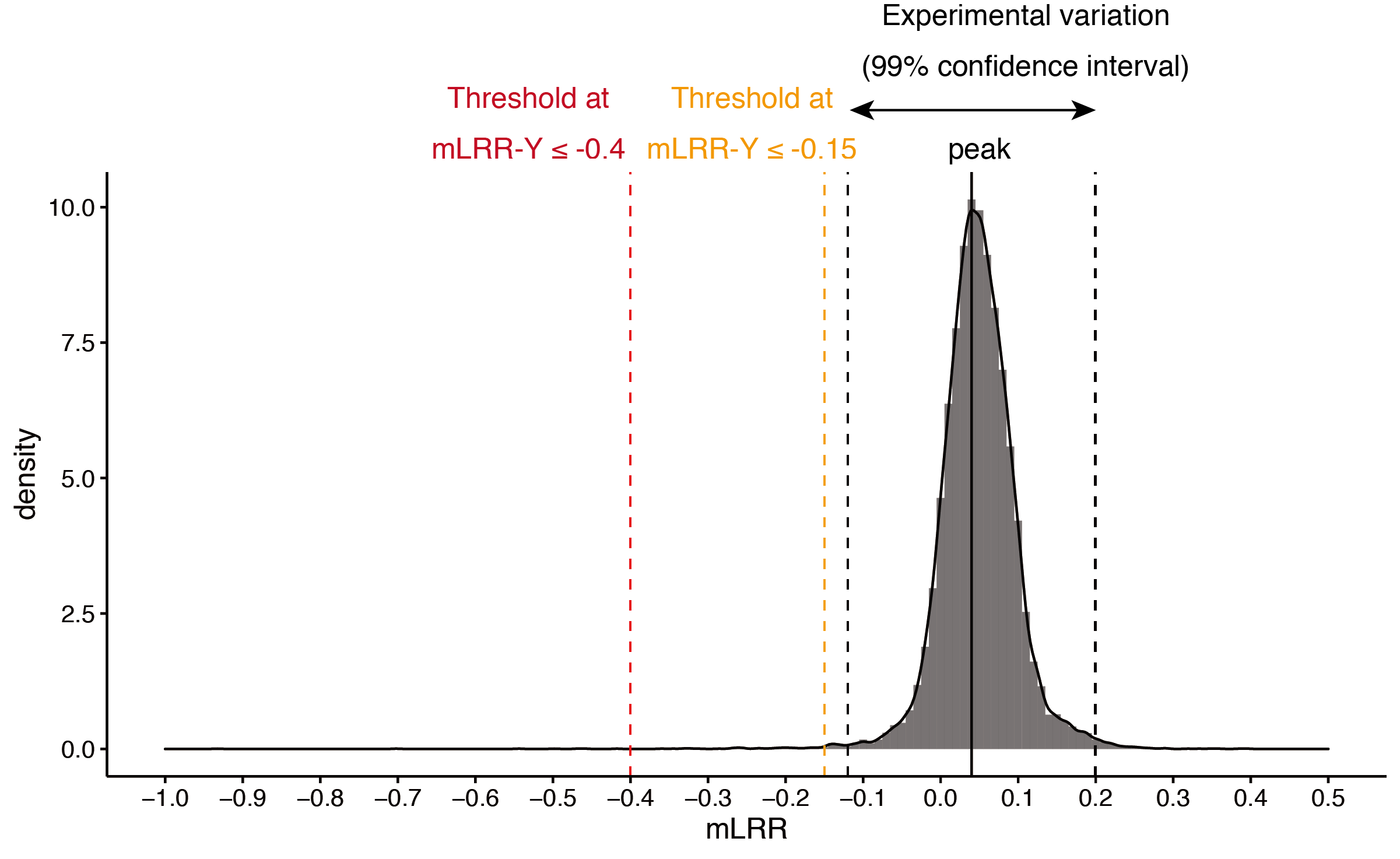
**

**Supplementary Figure 3. Distribution of mLRR for the chip version 4.2**

The *x*-axis indicates the median of log R ratios for probes in the male-specific region of chromosome Y (mLRR-Y). The *y*-axis shows the density of subjects genotyped by the Genesis Healthcare customized chip version 4.2 (*n* = 16,450). The number of markers in the male-specific region of chromosome Y was 2,623. The peak of the distribution is shown as a black vertical line, and ranges of experimental noise (99% confidence interval) are indicated by dashed black vertical lines. Thresholds at mLRR-Y ≤ -0.15 and ≤ -0.4 are shown as orange and red dashed lines, respectively. The threshold of mLRR-Y ≤ -0.15 was applied for all analyses in this study.

**Supplementary Figure 4. An exemplified plot of probe position and log R ratio values**

The *x*-axis indicates the probe position on the chromosome Y. The *y*-axis shows the log R ratio values for a subject genotyped by chip version 4.2. Data points within the male-specific region (MSR) was shown in blue, and other data points were shown in gray. The number of markers in the male-specific region (MSR) of chromosome Y was 2,623, whereas 181 probes were located outside the MSR. A red horizontal line indicates the median of log R ratio (-0.544).

MSR indicates male-specific region.

**Supplementary Table 1. Statistical power to detect significant difference between target and reference groups**

| Variable | Target group (*N* in this group) | Reference group (*N* in this group) | Number of subjects in reference group with mLOY | Assumed odds ratio | Statistical power |
| --- | --- | --- | --- | --- | --- |
| Smoking | Current  (1,812) | Never  (3,573) | 18 | 4.0 | 0.996 |
|  |  |  |  | 3.0 | 0.939 |
|  |  |  |  | 2.0 | 0.556 |
|  | Former  (2,891) | Never  (3,573) | 18 | 4.0 | 1.000 |
|  |  |  |  | 3.0 | 0.979 |
|  |  |  |  | 2.0 | 0.646 |
| Drinking frequency | 3–5  (1,506) | 1–2  (2,030) | 4 | 4.0 | 0.717 |
| (days per week) |  |  |  | 3.0 | 0.483 |
|  |  |  |  | 2.0 | 0.204 |
|  | ≥6  (1,795) | 1–2  (2,030) | 4 | 4.0 | 0.744 |
|  |  |  |  | 3.0 | 0.501 |
|  |  |  |  | 2.0 | 0.207 |
| Body mass index | <20.0  (2,447) | 20.0–24.9  (14,343) | 62 | 4.0 | 1.000 |
| (kg/m^2^) |  |  |  | 3.0 | 0.989 |
|  |  |  |  | 2.0 | 0.744 |
|  | ≥30  (1,597) | 20.0–24.9  (14,343) | 62 | 4.0 | 0.997 |
|  |  |  |  | 3.0 | 0.953 |
|  |  |  |  | 2.0 | 0.621 |
| Physical activity | ≥6  (425) | <1  (3,817) | 18 | 4.0 | 0.820 |
| (days per week) |  |  |  | 3.0 | 0.621 |
|  |  |  |  | 2.0 | 0.300 |

mLOY indicates mosaic loss of the Y chromosome

**Supplementary Table 2. Number of study participants and prevalence of mLOY according to age group**

| Age group (years) | *N* | Number of subjects with mLOY | Prevalence |
| --- | --- | --- | --- |
|  |  |  |  |
| 18–24 | 1,212 | 2 | 0.0017 |
| 25–29 | 2,628 | 3 | 0.0011 |
| 30–34 | 3,705 | 8 | 0.0022 |
| 35–39 | 3,858 | 12 | 0.0031 |
| 40–44 | 3,978 | 5 | 0.0013 |
| 45–49 | 3,490 | 4 | 0.0011 |
| 50–54 | 2,614 | 11 | 0.0042 |
| 55–59 | 1,765 | 10 | 0.0057 |
| 60–64 | 1,032 | 8 | 0.0078 |
| 65–69 | 525 | 10 | 0.0190 |
| 70–74 | 214 | 11 | 0.0514 |
| 75–79 | 61 | 3 | 0.0492 |
| 80–84 | 23 | 4 | 0.1739 |
| 85–89 | 9 | 2 | 0.2222 |
| 90–93 | 7 | 3 | 0.4286 |

mLOY indicates mosaic loss of the Y chromosome

**Supplementary Table 3. Association between physical activity and mLOY according to age group**

| Age group (years) | Physical activity  (days per week) | *N* | Number of subjects with mLOY | Prevalence | Odds ratio  (95% CI) | *P* |
| --- | --- | --- | --- | --- | --- | --- |
| <40 | <1 | 1,760 | 7 | 0.0040 | Reference | – |
|  | 1–2 | 1,421 | 3 | 0.0021 | 0.54 (0.12–1.96) | 0.38 |
|  | 3–5 | 487 | 3 | 0.0062 | 1.63 (0.35–5.93) | 0.48 |
|  | ≥6 | 195 | 1 | 0.0051 | 1.36 (0.07–7.82) | 0.78 |
| 40–49 | <1 | 1,195 | 2 | 0.0017 | Reference | – |
|  | 1–2 | 866 | 2 | 0.0023 | 1.38 (0.17–11.53) | 0.75 |
|  | 3–5 | 331 | 2 | 0.0060 | 3.63 (0.43–30.33) | 0.20 |
|  | ≥6 | 117 | 1 | 0.0085 | 5.17 (0.24–54.63) | 0.18 |
| 50–59 | <1 | 631 | 7 | 0.0111 | Reference | – |
|  | 1–2 | 459 | 2 | 0.0044 | 0.39 (0.06–1.63) | 0.24 |
|  | 3–5 | 185 | 1 | 0.0054 | 0.48 (0.03–2.72) | 0.49 |
|  | ≥6 | 65 | 2 | 0.0308 | 2.85 (0.42–12.10) | 0.20 |
| ≥60 | <1 | 231 | 2 | 0.0087 | Reference | – |
|  | 1–2 | 182 | 3 | 0.0165 | 6.86 (0.71–184.04) | 0.14 |
|  | 3–5 | 105 | 1 | 0.0095 | 1.47 (0.06–22.24) | 0.78 |
|  | ≥6 | 48 | 3 | 0.0625 | 21.02 (2.12–571.17) | 0.02 |
| ALL | <1 | 3,817 | 18 | 0.0047 | Reference | – |
| (18–93) | 1–2 | 2,928 | 10 | 0.0034 | 0.80 (0.35–1.72) | 0.57 |
|  | 3–5 | 1,108 | 7 | 0.0063 | 1.26 (0.48–2.95) | 0.61 |
|  | ≥6 | 425 | 7 | 0.0165 | 3.45 (1.30–8.21) | 0.007 |

All association analyses (odds ratios and *P*-values) shown in this table were performed using a logistic regression model adjusted for 15 age group categories (18–24, 25–29, 30–34, 35–39, 40–44, 45–49, 50–54, 55–59, 60–64, 65–69, 70–74, 75–79, 80–84, 85–89, or 90–93 years).

CI indicates confidence interval; mLOY, mosaic loss of the Y chromosome

**Supplementary Table 4. Statistical power for the association analysis between mLOY and risk of disease**

| Outcome | Number of subjects without disease history | Number of subjects with disease history | Statistical power |
| --- | --- | --- | --- |
|  |  |  |  |
| Cancer | 19,906 | 300 | 0.258 |
| Diabetes mellitus | 19,710 | 489 | 0.325 |
| Cardiovascular disease | 19,743 | 455 | 0.318 |

Statistical power to detect a significant difference between subjects with and without medical history are shown. A modest effect (odds ratio of 2.0) was assumed. Self-reported medical history was collected using a web-based questionnaire.

mLOY indicates mosaic loss of the Y chromosome
